# Long non-coding RNAs: emerging players regulating plant abiotic stress response and adaptation

**DOI:** 10.1186/s12870-020-02595-x

**Published:** 2020-10-12

**Authors:** Uday Chand Jha, Harsh Nayyar, Rintu Jha, Muhammad Khurshid, Meiliang Zhou, Nitin Mantri, Kadambot H. M. Siddique

**Affiliations:** 1 ICAR-Indian Institute of Pulses Research (IIPR), Kanpur, 208024 India; 2 Department of Botany, Panjab University, Chandigarh, India; 3 Institute of Crop Sciences, Chinese Academy of Agricultural Sciences, Beijing, China; 4 Institute of Biochemistry and Biotechnology, University of the Punjab, Lahore, Pakistan; 5 School of Science, RMIT University, Plenty Road, Bundoora. Victoria. 3083., Australia; 6 The UWA Institute of Agriculture, The University of Western Australia, Perth, WA 6001 Australia

**Keywords:** Abiotic stresses, Long non-coding RNAs, Gene regulation, Target mimicry

## Abstract

**Background:**

The immobile nature of plants means that they can be frequently confronted by various biotic and abiotic stresses during their lifecycle. Among the various abiotic stresses, water stress, temperature extremities, salinity, and heavy metal toxicity are the major abiotic stresses challenging overall plant growth. Plants have evolved complex molecular mechanisms to adapt under the given abiotic stresses. Long non-coding RNAs (lncRNAs)—a diverse class of RNAs that contain > 200 nucleotides(nt)—play an essential role in plant adaptation to various abiotic stresses.

**Results:**

LncRNAs play a significant role as ‘biological regulators’ for various developmental processes and biotic and abiotic stress responses in animals and plants at the transcription, post-transcription, and epigenetic level, targeting various stress-responsive mRNAs, regulatory gene(s) encoding transcription factors, and numerous microRNAs (miRNAs) that regulate the expression of different genes. However, the mechanistic role of lncRNAs at the molecular level, and possible target gene(s) contributing to plant abiotic stress response and adaptation, remain largely unknown. Here, we review various types of lncRNAs found in different plant species, with a focus on understanding the complex molecular mechanisms that contribute to abiotic stress tolerance in plants. We start by discussing the biogenesis, type and function, phylogenetic relationships, and sequence conservation of lncRNAs. Next, we review the role of lncRNAs controlling various abiotic stresses, including drought, heat, cold, heavy metal toxicity, and nutrient deficiency, with relevant examples from various plant species. Lastly, we briefly discuss the various lncRNA databases and the role of bioinformatics for predicting the structural and functional annotation of novel lncRNAs.

**Conclusions:**

Understanding the intricate molecular mechanisms of stress-responsive lncRNAs is in its infancy. The availability of a comprehensive atlas of lncRNAs across whole genomes in crop plants, coupled with a comprehensive understanding of the complex molecular mechanisms that regulate various abiotic stress responses, will enable us to use lncRNAs as potential biomarkers for tailoring abiotic stress-tolerant plants in the future.

## Background

The immobile nature of plants means that they can be frequently confronted by various biotic and abiotic stresses during their lifecycle. Plants have evolved several complex mechanisms to recognize various stress factors, generate appropriate signaling pathways, and respond accordingly by reprogramming the expression of multiple genes at the transcriptional, post-transcriptional, and epigenome level to adapt under harsh environment conditions [1, 2]. The research community has successfully identified several complex mechanisms that plants use at the genetic, physiological, biochemical, and molecular levels to maintain ‘cellular homeostasis’ under unfavorable environments [2, 3]. The discovery of miRNAs (21–24 nt)—a novel class of non-coding RNAs (ncRNAs)—and their regulatory mechanisms for controlling genes involved in various developmental, biological, and stress responses has advanced our understanding of gene regulation in plants [4, 5]. The technical innovations of genome sequencing, especially next-generation sequencing, RNA-sequencing (RNA-seq), and advanced bioinformatics tools, have improved the functional elucidation of various genes at the transcription, post-transcription, post-translation, and epigenetic level [6]. These innovations have enabled the discovery of novel ncRNAs, including lncRNAs, and their role in regulating various biological processes, development, and stress responses in mammals and plants (for details, see [7, 8]). LncRNAs are a diverse class of RNAs, and the largest class acting as ‘biological regulators’ that control transcriptional regulation and genome imprinting [9, 10]. Numerous noteworthy instances of lncRNAs regulating plant development, disease resistance, nutrient acquisition, and other biological processes through chromatin remodeling, histone modification, pri-mRNA alternative splicing, or acting as ‘target mimicry’ have been recorded [11–15]. However, few studies have undertaken genome-wide exploration of lncRNAs, their complex regulatory molecular mechanisms, or functional annotation [16]. Here, we explain the types and functions of lncRNAs and update the roles of various lncRNAs, their target gene(s), and the complex operational molecular mechanisms involved in acclimating plants to the challenging environments of various abiotic stresses.

### Biogenesis, type, and functions of lncRNAs

**Fig. 1:**
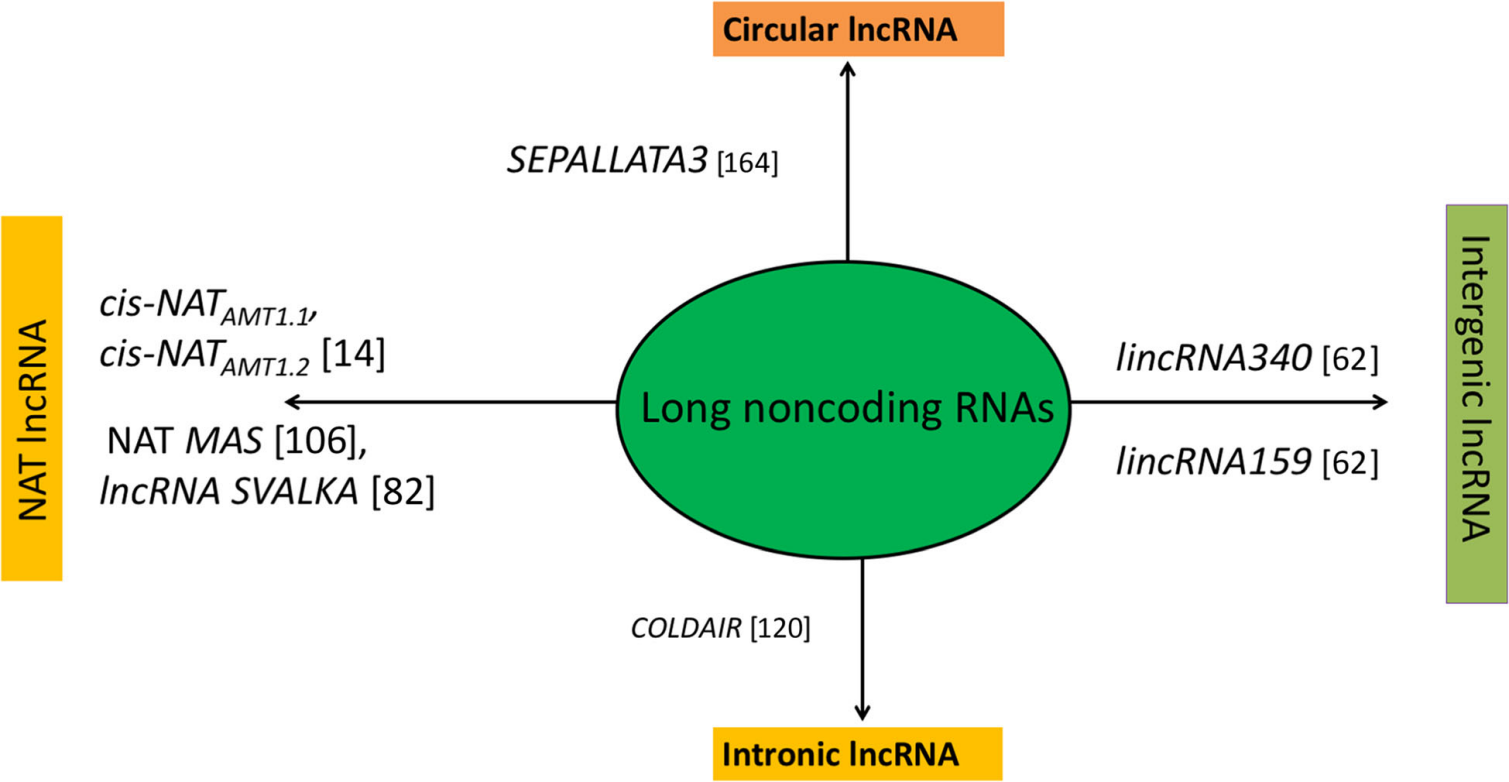
Various types of lncRNAs with suitable examples in various plants [164]

**Fig. 2:**
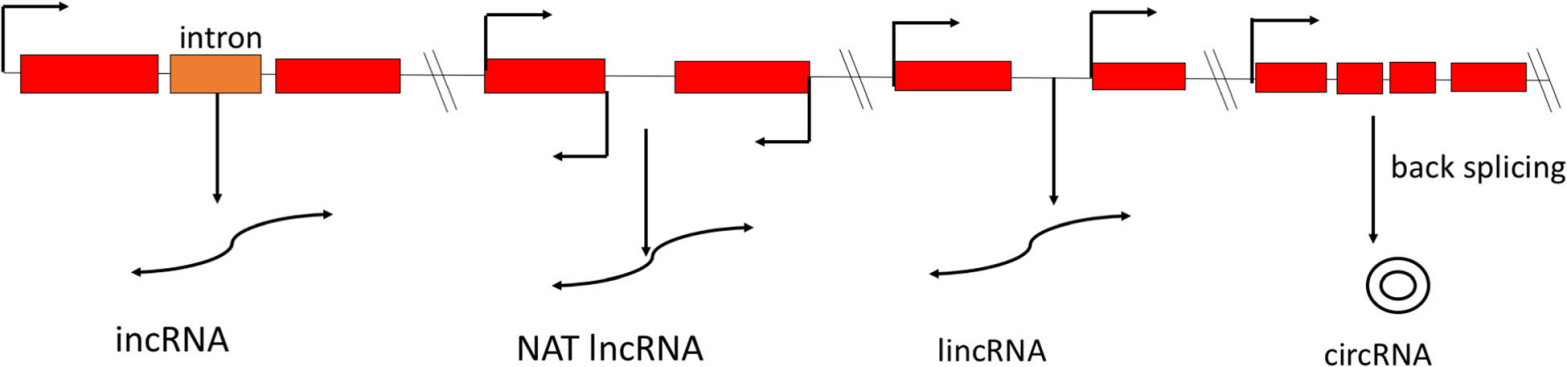
Biogenesis of various lncRNAs. LincRNAs originate from intergenic regions, while incRNAs are transcribed from intronic regions. NATs originate from complementary DNA strands of sense coding regions [26], while circRNAs originate from a ‘back-splicing reaction of internal exons in pre-mRNA’ [29, 31]. The figure is modified from Wang and Chekanova [33] and Wu et al. [29]

Among the various classes of ncRNAs, lncRNAs are a heterogeneous class of RNA transcripts > 200 nt that are incapable of coding proteins, act as ‘riboregulators,’ are located in the nucleus or cytoplasm, and are transcribed by RNA polymerase II or III and polymerase IV/V [17–19]. Pol IV lncRNAs serve as precursors for small interfering RNAs (siRNAs) [19]. Pol V-dependent lncRNAs assist in modulating the local chromatin loop [20], are transcribed from either strand of the protein-coding locus, may or may not have 5´ cap and poly-adenylation at 3´ tail, and are expressed in a ‘tissue-specific’ manner [21–23]. LncRNAs can be broadly classified as (i) long intergenic ncRNAs (lincRNAs), (ii) intronic ncRNAs (incRNAs), (iii) natural antisense transcripts (NATs), and (iv) circular long non-coding RNAs (circRNAs) based on their location and neighboring protein-coding genes [22, 24–26]. LincRNAs originate from intergenic regions, featuring weakly spliced, polyadenylated tissue-specific expression, and execute *trans* (distant gene) regulatory function [27–29], while incRNAs are transcribed from intronic regions. NATs originate from complementary DNA strands of sense coding regions [26] and feature *cis-* and *trans*-regulatory action [30]. However, circRNAs are in low abundance, originate from the ‘back-splicing reaction of internal exons in pre-mRNA’ [29, 31], feature a covalently closed structure, and display higher sequence conservation than linear lncRNAs [29, 32]. Various types of lncRNAs and their possible biogenesis are illustrated in Figs. 1 and 2. They act functionally as ‘decoy’ or ‘sponge molecules,’ ‘signal molecules,’ ‘backbone molecules,’ and ‘guide molecules’ [25, 34, 35]. Moreover, lncRNAs can be precursors of miRNAs and siRNAs, regulate alternative splicing of pre-mRNAs, and serve as endogenous target mimics (eTM) competing for various miRNAs [20, 36, 37].

### Sequence conservation, diversity and phylogenetic features of plant lncRNAs

The highly evolved nature of lncRNAs has resulted in lower sequence conservation across plant and animal species and, thus, fewer phylogenetic relationships [38, 39]. Marques and Ponting [40] reported that < 2% of lncRNAs in *Arabidopsis thaliana* were evolutionarily conserved across the plant kingdom, which explains the rapid evolution of lncRNA sequences. Conservation analysis of lncRNAs from five monocot and five dicot species demonstrated high sequence conservation at the intra-species and sub-species level [41]. At the interspecific level, lncRNAs remain highly diverged at the nucleotide level and have shown a diverse regulatory role [41, 42]. Mohammadin et al. [43] also supported positional sequence conservation of lncRNAs in *Aethionema arabicum* and *Tarenaya hassleriana* at the nucleotide level using a phylogenomics approach. Likewise, Golicz et al. [44] confirmed the sequence homology of four lncRNAs in soybean, chickpea, and *Medicago truncatula.* Despite sequence dissimilarity, lncRNAs were similar in terms of their low expression capability, short length, and fewer exons and splice variants across numerous plant species, including *Arabidopsis*, cucumber, maize, chickpea, and soybean [43–47]. Likewise, the conserved function of lncRNAs in both animal and plant species has been investigated [38]. The growing database of lncRNAs and comparative genomics analyses could provide new impetus into the functional conservation of lncRNA genes and their modes of action and function across various plant species [38].

### lncRNAs controlling drought stress tolerance

**Fig. 3:**
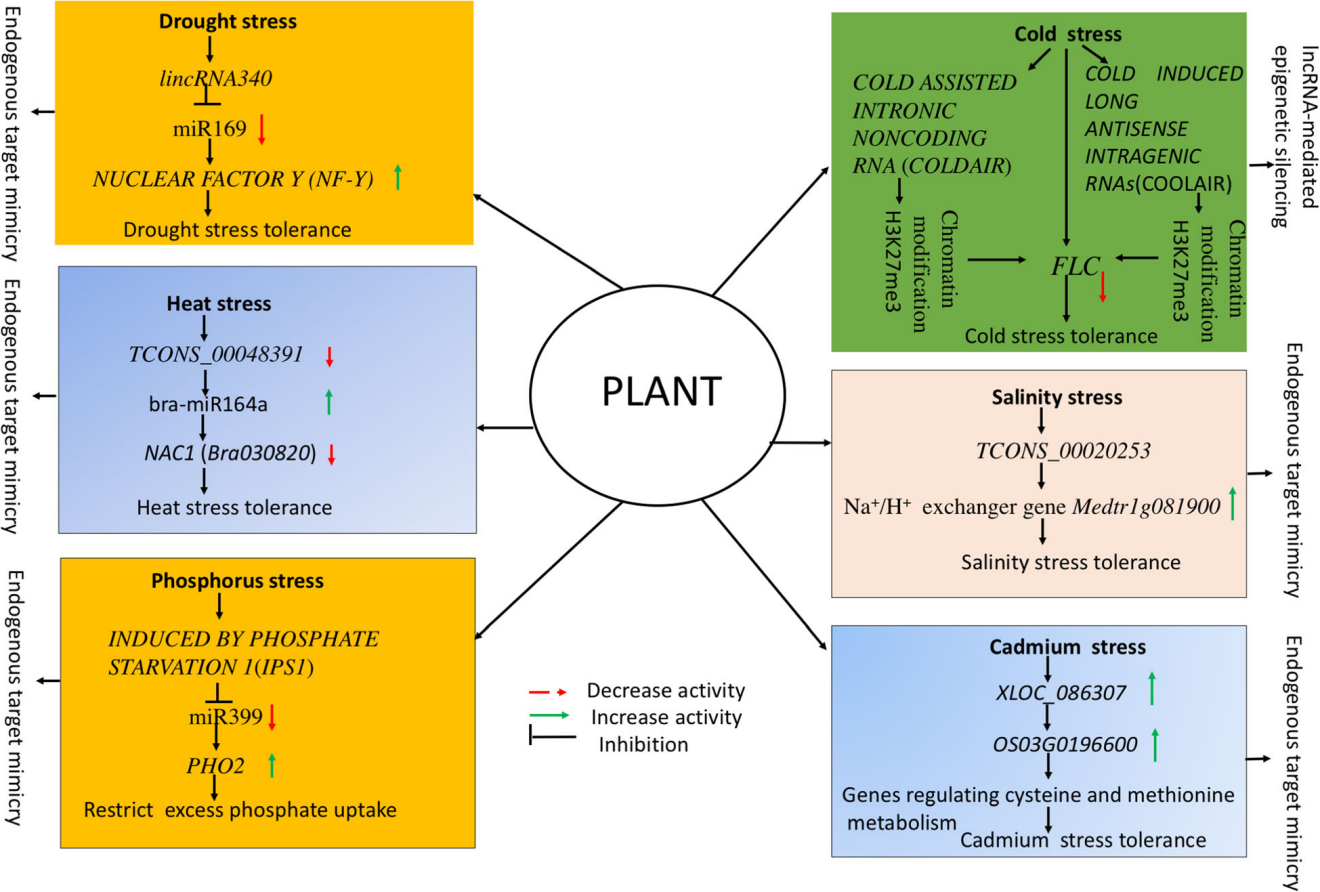
LncRNA module controlling environmental abiotic stress response in plants. Relying on the target mimic mechanism increased the expression of *lincRNA340* under drought, which reduced the activity of target miR169 and ultimately increased *NUCLEAR FACTOR Y (NF-Y)* gene expression [60]. The lncRNAs *TCONS_00048391* and *TCONS_00010856* acted as endogenous target mimics for bra-miR164a, which regulates the heat stress response [64]. Repression of the *FLC* locus during the early onset of cold stress is controlled by *COLD INDUCED LONG ANTISENSE INTRAGENIC RNAs* (COOLAIR), an alternatively spliced NAT lncRNA transcribed from the antisense orientation of *FLC* gene by chromatin modification (reducing active histone mark H3K36me3 and enhancing repressive histone mark H3K27me3) of the *FLC* locus during vernalization [65]. Under salinity stress *TCONS_00020253 up-regulate expression of* Na^+^/H^+^ exchanger gene *Medtr1g081900* in roots [68]. Inductive mechanism of lncRNA *INDUCED BY PHOSPHATE STARVATION 1*(*IPS1*) works as an eTM or decoy for miR399 and regulates the target *PHO2* gene expression and phosphate homeostasis in *Arabidopsis* (Franco-Zorrilla et al. [11] and Bari et al. [69]). Significant up-regulatory activity of lncXLOC_086307 suggests that XLOC_086307 likely participates in Cd response processes in rice by controlling the cysteine-rich peptide metabolism-related gene *OS03G0196600* [88]

Globally, episodes of drought stress-related events are increasing due to the erratic pattern of rainfall, which affects plant growth and poses a serious challenge for global food security [48]. Plants have a variety of physiological, biochemical, and complex molecular networks, including cascades of various signal transduction pathways, to adapt under drought stress [49]. Advances in molecular biology have uncovered the underlying gene(s)/QTLs and various complex regulatory gene networks and molecular signaling cascades controlling the drought stress response in plants [48, 50]. Subsequently, the discovery of drought-responsive miRNAs and their candidate target genes in various plants has shed light on the molecular mechanisms involved in drought stress adaptation (see [51]). Likewise, emerging evidence has revealed a participatory role of lncRNAs in response to drought stress in plants, capitalizing on the co-expression network based on lncRNAs, miRNAs and protein-coding genes, and transcription factors [52–54]. Notable instances of drought-responsive lncRNAs have been reported in various plant species—six in *Arabidopsis* [55], 504 in *Populus* spp. [56], 98 in rice [57], 664 in maize [58], 19 in foxtail millet [59], 185 in cassava [60], and 1597 in switchgrass [52]. LncRNAs could affect the drought stress response by recruiting complex mechanisms based on eTM, antisense transcription-mediated modulation, chromatin modulation, or directly regulating the transcription of various drought-responsive genes [60–63]. Deep sequencing of foxtail millet provided an opportunity to explore 584 lncRNAs [59], of which 17 lincRNAs and two NAT lncRNAs exhibited differential expression under drought stress. Concurrently, the authors found 20 similar lincRNAs and one NAT lncRNA responding to drought stress in sorghum [59]. Only one drought-responsive lncRNA in foxtail millet exhibited sequence co-linearity with the drought-responsive lncRNA in sorghum, demonstrating the low conserved nature of lncRNAs [59]. In *Populus trichocarpa,* a systematic RNA-seq analysis explored a comprehensive landscape of > 2500 lncRNAs [56], of which 504 were drought-responsive. Functional validation of eight drought-responsive lncRNAs from the 504 drought-responsive lncRNAs using RT-qPCR revealed the up-regulation of six lincRNAs and down-regulation of two lincRNAs under water stress. To survey drought-responsive lncRNAs in the cassava genome, strand-specific RNA-seq data served to identify a set of 318 lncRNAs and 153 NAT lncRNAs responding to cold and drought stress [60]. Of the 51 drought-specific differentially expressed lncRNAs (DElncRNAs), 40 showed up-regulatory action under drought stress. Functional validation of selected lincRNAs using qRT-PCR revealed the up-regulation of *lincRNA101, lincRNA391,* and *lincRNA356* and down-regulation of *lincRNA64, lincRNA350, lincRNA182,* and *lincRNA392* under drought stress. Furthermore, relying on the target mimic mechanism increased the expression of *lincRNA340* under drought, which reduced the activity of target miR169 and ultimately increased *NUCLEAR FACTOR Y (NF-Y)* gene expression [60] see Fig. 3]. Ding et al. [53] recovered 124 DElncRNAs under drought stress in cassava, of which 11 worked as target mimics for miR156, miR164, miR169, and miR172. Functional validation revealed that *TCONS_00068353* lncRNA acted as a target mimic for miR156k and miR172c that control various abiotic stress-responsive genes, while *TCONS_00060863* and *TCONS_00097416* lncRNAs participated in the ABA and ethylene signaling pathways, respectively, under drought stress [53].

Considering the regulatory mechanism of NAT lncRNA, 98 drought-responsive lncRNAs were recovered in rice using RNA-seq analysis, along with two important drought-responsive lncRNAs *NAT Os02g0250700–01* (targeting *late embryogenesis abundant protein* gene) and *NAT Os02g0180800–01* (targeting *cinnamoyl-CoA reductase* gene) [57]. The expression of these two lncRNAs and their corresponding target genes remained inversely correlated. A study on genome-wide drought-responsive lncRNAs in maize identified 1535 lncRNAs at various developmental stages [54]. The lncRNAs captured at the R1 stage (silking stage) had a critical role in drought stress tolerance. Furthermore, the V-ATPase encoding gene (*vpp4*) was unearthed as a possible target gene for lncRNA*MSTRG.6838.1*; *vpp4* and the identified lncRNA may work as cis-acting pairs.

Apart from acting as eTM or NAT, lncRNAs could regulate the transcription of various drought stress-responsive genes [52, 62]. The possible role of lncRNAs in regulating drought stress tolerance has been explored in *Arabidopsis*, with the identification of a novel lncRNA *DROUGHT INDUCED lncRNA* (*DRIR*) localized in the nucleus, containing a 755 nt long lincRNA that controls several drought stress-responsive genes, including ABA-signaling genes (*ABI5, P5CS1, RD29A*, and *RD29B*), aquaporin genes (*NIP1*, *TIP4*), annexin gene *(ANNAT7)*, *FUCOSYL TRANSFERASE4* (*FUT4*) gene, and transcription factor genes (*NAC3, WARKY8*) at the transcription level [37]. The *drir*^*D*^(T-DNA insertion mutant) and *DRIR*-overexpressing *Arabidopsis* lines had higher drought tolerance than wild-type seedlings, as revealed in the higher-fold expression of these genes. Thus, the lncRNA *DRIR* conferred water-deficit stress tolerance by serving as a positive regulator.

Likewise, lncRNAs regulating various drought-responsive regulatory genes participating in ethylene and ABA synthesis and signaling, calcium signaling, starch and sucrose synthesis, and various metabolic processes have been reported in rice [63], switchgrass (*Panicum virgatum* L.) [52], *P*. *betulifolia* [70], cassava [53, 71], and *Cleistogenes songorica* [62] (see Table 1). Of the 441 DElncRNAs identified in switchgrass under drought stress imposed at various growth stages, lncRNAs *XLOC_053020*, *XLOC_014465*, and *XLOC_033252* controlling ABA synthesis, *XLOC_074836* contributing to ethylene signaling, and *XLOC_005809* involved in trehalose phosphate synthase metabolism were up-regulated, suggesting their significant role in drought- stress tolerance [52]. Various lncRNAs and their possible target gene(s) and working mechanisms contributing to drought stress and other abiotic stress responses in various crops have been identified (see Table 2). Collectively, the various lncRNAs play a role in controlling drought stress by acting as target mimics for various miRNAs that control the expression of various drought-responsive target genes or transcription factors through up- or down-regulation. These emerging lncRNAs could act as a regulatory hub for controlling various drought-responsive hormonal signaling pathways at the transcription, post-transcription, and epigenome level.

**Table 1:** Various types of lncRNAs that control abiotic stress responses in plants

Name of stress	Crop	No. of lncRNAs identified	Number of lncRNAs expressed under stress	Platform and technique used for lncRNAs identification and their function	Function	Reference
Drought	Foxtail millet	19 lncRNAs	19	IlluminaHiSeq 2000,qRT-PCR	Control drought stress response	[59]
Drought	*Populus trichocarpa*	2542 lincRNAs	504	HiSeq™ 2000, RT-qPCR	Drought- stress response	[56]
Drought	Rice	98 lncRNAs	98	Illumina HiSeq 2500, qRT-PCR	Regulatory role in drought response	[57]
Drought	*Arabidopsis*	*DROUGHT INDUCED lncRNA (DRIR)*	*DROUGHT INDUCED lncRNA (DRIR)*	HiSeq 2000, RT-qPCR	Participate in regulating set of drought responsive genes	[37]
Drought	Rice	3714	21	RT-qPCR, PLncPRO	Differentially expressed under drought stress	[72]
Drought	Wheat	–	59,110	Illumina HiSeq. 2000, qRT-PCR	Differential expression under drought stress response	[73]
Drought and cold	Cassava	682 lncRNAs	318	HiSeq 2500,qRT-PCR, CNCI, CPC,	Hormone signal transduction, sucrose metabolism pathway etc.	[60]
Drought	*Pyrus betulifolia*	14,478	251	Illumina HiSeq 4000, CNCI, CPC, qRT-PCR	Various metabolic processes	[70]
Drought	*Panicum virgatum L*	16,551 novel lncRNAs	1597	HiSeq2500, qRT-PCR	Regulating drought-stress response	[52]
Drought	Maize	3488	1535	Illumina HiSeq 2500, qRT-PCR	Oxidoreductase activity, water binding, and electron carrier activity	[54]
Drought	*Cleistogenes songorica*	3397 lncRNAs	468	HiSeq2500, CPC, CNCI, CPATqRT-PCR	Regulating drought-stress response	[62]
Drought	Cassava	833	124	Hiseq 4000, qRT-PCR, CNCI, CPC,	Cell-related metabolism, Calvin cycle, hormone metabolism etc.	[53]
Drought	Cassava	1405	185	qRT-PCR	Melatonin responsive controlling drought-stress response	[74]
Drought	Cassava	1379	194	qRT-PCR	ABA signaling regulation	[71]
Heat stress	Wheat	125 putative	77	Solexa sequencing technology wheat Affymetrix GeneChip, qRT-PCR	Heat responsive	[75]
Heat stress	*Brassica rapa ssp. chinensis*	4594 putative lncRNAs	1686	Illumina Hiseq. 2500, qRT-PCR CPC,CNCI	Differential expression of these RNA suggested involvement of various phytohormones in heat stress tolerance.	[64]
Heat stress and drought	*Brassica juncea*	7613 putative lncRNAs	1614	qRT-PCR	Associated with enzymatic and non-enzymatic antioxidants under drought and heat stress	[76]
Cold and heat	Chinease cabbage	10,001	2236	Illumina HiSeq™ 2000 qRT-PCR, CPC	Total of 67 and 192 target genes for cold and heat were regulated	[77]
Cold stress	Banana	12,462 lncRNAs	20	Illumina HiSeqTM 4000, qPCR, CPC	Cold stress response	[78]
Cold stress	*Arabidopsis*	379	135	Illumina HiSeq 2500, RT-qPCR	Cold or freezing acclimation	[79]
Cold stress	*Arabidopsis*	*SVALKA*	*SVALKA*		Repress *CBF1* expression and freezing tolerance	[80]
Cold stress	Grapevine	2088	466	HiSeq 2500, qRT-PCR, CNCI, CPC,	Related to cold stress response	[81]
Cold stress	Chinese cabbage	2088	549	Illumina HiSeqTM 2000, qPCR	Controlling vernalization	[82]
Cold stress	Rice	1485 lncRNAs	566	Illumina HiSeq 2500 platform, qRT-PCR	Controlling cold stress response	[83]
Cold stress	*Medicago*					
	*truncatula*	24,368 unique lncRNAs	983 and 1288	Illumina HiSeq 4000,Q-PCR	Controlling cold stress response	[84]
Salinity	*Arabidopsis*	*DROUGHT INDUCED lncRNA (DRIR)*	*DROUGHT INDUCED lncRNA (DRIR)*	HiSeq 2000, RT-qPCR	Participate in regulating set of salinity responsive genes	[37]
Salinity and drought	Chickpea	3457	13	RT-qPCR, PLncPRO	Differentially expressed under drought and salinity stress	[72]
Salinity	Barley	*CNT0018772* and *CNT0031477*	*2*	qPCR	Both up- and down- regulatory role in salinity stress	[85]
Salinity	Cotton	1117 unique lncRNAs	44	Illumina HiSeq 4000, RT-qPCR	Controls salinity stress genes	[86]
Salinity and boron	Maize	48,345	1710	Illumina MiSeq, RT-qPCR, AgriGO	Nicotianamine biosynthetic and metabolic processes, gene regulation	[87]
Salinity	Poplar	10,646 and 10,531 lncRNAs	8592 and 3425	HiSeq 2500	Regulating *osmotin 34*, *NHX7*, RARE-COLD-*INDUCIBLE 2B,* and *WRKY 33 genes*	
Cadmium stress	Rice	3558	69 lncRNAs were up-regulated and 75 lncRNAs were down-regulated	Illumina HiSeq 2000,CPC, RT-qPCR	Genes related to phtosynthetic pathways are involved in response to Cd stress	[88]
and salinity	Wheat	44,698	2064 and 2278		Regulatory roles in numerous biological processes	[89]
Ca^2+^-channel blocker	Wheat	6309	177	HiSeqTM2000, qRT-PCR	Affects various biological processes	[90]
Oxidative stress	Rice	7000 lncRNAs		Hiseq2000, DEGSeq	Down-regulated poly adenylation lncRNAs participate in abiotic stress tolerance	[91]
Waterlogging	Maize	6099	3190	Illumina HisSeq 4000, qRT-PCR	Metabolic pathways, such as glycolysis and methionine metabolism in response to water logging	[92]
Phosphate starvation	Arabidopsis	1212 novel lncRNAs	309	Illumina Hiseq 2000/2500,	Phosphate starvation signaling and regulation	[93]
				qRT-PCR	Cell wall organization and photosynthesis	
Phosphate deficiency	*Medicago truncatula*	10, 785	358 and 224	Illumina Hiseq2000, qRT-PCRCPC,CNCI	Involved in various signal transduction, chemical detoxification	[61]
Phosphorus use efficiency	barley	188 and 209	–	Illumina sequencing, qRT-PCR	Related to phosphate starvation	[94]
Nitrogen deficiency	Poplar	388	126		Low nutrition adaptation	[95]
Nitrogen deficiency	Maize	7245	637	Illumina HiSeq™2500,CPC,qPCR	Nitrogen metabolism, oxidative phosphorylation	[96]
Nitrogen deficiency	Rice	2588 novel putative lncRNA	2588	Illumina HiSeq 2500, qRT-PCR	Regulatory role in N-starvation- response	[14]
Nitrogen deficiency	Barley	498 lncRNAs	56	Illumina Hiseq Xten platform	Regulatory role in N-starvation- response	[97]
				qPCR		[98]
Boron deficiency	*Poncirus*	2101 unique lncRNAs		Illumina HiSeq X Ten platform	Regulatory role in B-starvation response	
	*trifoliata*			qRT-PCR		
Low nutrient deficiency	Arabidopsis	60 differentially expressed lincRNAs	60 differentially expressed lincRNAs	HiSeq2000TM, qRT-PCR	Controlling various nutrient response	[99]
CPC=Coding Potential Calculator						
CNCI=Coding-Non-Coding Index						
CPAT = Coding Potential Assessment Tool

**Table 2:** Function of various lncRNAs regulating various abiotic stress in plants

Stress	Crop	Genotype	LncRNA	Target gene	Regulatory mechanism	Reference
Drought	*Populus*	Nisqually 1	lincRNA20 and lincRNA2752	**–**	Control drought stress by regulating	[56]
	*trichocarpa*		lincRNA2962 and lincRNA1039		ptc-miR476 and ptc-miR169 through eTM	
			LincRNA3241			
Drought	Rice	*Oryza sativa* cv. Ilmi	*NAT Os02g0250700–01*	*Os02g0250600–01*	Regulate drought by NAT lncRNAs	[57]
			*NAT Os02g0180800–01*	(late embryogenesis		
				abundant protein)		
				*Os02g0180700–01*		
				(cinnamoyl-CoA		
				reductase)		
Drought	Rice	DXWR	Up-regulated lncRNAs MSTRG69391	Transcription factor, calmodulin	Regulate biological processes in	[63]
			MSTRG41712 and MSTRG68635 and	HSP genes, mitochondrial carrier	response to drought stress	
			down regulated lncRNAs MSTRG65848	protein gene etc		
			MSTRG27834 and MSTRG46301			
Drought	Cassava	TMS60444	*lincRNA340*	*NUCLEAR FACTOR Y (NF-Y)*	By targeting miR169 based on target mimicry	[60]
Drought	Wheat	Kiziltan and TR39477	*c70772_g2_i1 and c90557_g1_i1*	*c69036_g1_i1* and	Drought stress is regulated by	[73]
		TTD-22		c9653_g1_i2	lncRNA-miRNA-mRNA networks	
Drought	*Panicum*	Alamo	*XLOC_053020*	*Pavir.Ia01153*	Regulation of genes related	[52]
	*virgatum* L		*XLOC_014465*	*Pavir.Bb00347*	to ethylene synthesis	
			*XLOC_033252*	*Pavir.Eb01847*	and signaling, ABA synthesis and signaling,	
			*XLOC_090250, XLOC_016922,*	Pavir.J23169 and	starch and sucrose biosynthesis gene	
			and XLOC_067866	Pavir.Ca01179		
			*XLOC_074836*	*Pavir.J04626*		
			*XLOC_008122*	*Pavir.J10665*		
			*XLOC_081155*	Pavir.Ba00729		
			*XLOC_005809*	Pavir.Ab03141		
Drought	Cassava	Ku50	*TCONS_00060863, TCONS_00068353*	*CYP707A1*	Genes involved in ABA catabolism,	[53]
			*TCONS_00097416, TCONS_00069665,*	*CSLD5, ERL1, SPCH,*	ethylene signaling.	
				*LAX2, HDG11,SCR*	Also regulates gene by targeting miR156,	
			*TCONS_00040721*	*GRF1* and *HB51,DOX1*	miR164, miR169, and miR172	
Drought	*Cleistogenes*		*MSTRG.43964.1*	Genes related to	By regulating miRNA166, miRNA164, miRNA393, and miRNA397a/b and acting as endogenous target mimics	[62]
	*songorica*		*MSTRG.4400.2*	abscisic acid (ABA)		
				signalling pathway,		
			*MSTRG.42613.1*	Genes related to starch		
			*MSTRG.25585.13*	and sucrose metabolism		
Drought	Maize	B73	*lncRNA MSTRG6838.1*	V-ATPase encoding gene,	lncRNA regulating transcriptional	[54]
				*vpp4*	regulation by *cis*- and *trans*-acting modes	
Drought	Cassava		*TCONS_00129136, TCONS_00122745*			[71]
			TCONS_00088201,TCONS_00067612			
Drought	Cassava		*TCONS_00003360, TCONS_00015102*		Calcium signaling, ABA and	[74]
			*TCONS_00149293*		ethylene metabolism	
Drought	*Brassica napus*	Q2 and Qinyou8	*XLOC_042431, XLOC_071559,*	*BnaC06g05090D*	IAA, Cytokinin and ABA signalling	[100]
			*XLOC_ 095305, XLOC_100682,*	*BnaA01g17750D*	alpha-trehalose-phosphate synthase	
			*XLOC_019521 and XLOC_ 042894*	*BnaC07g44670D*		
			*XLOC_075476 and XLOC_074677,*	*BnaC02g25020D, BnaC02g25150D,*		
			*XLOC_074677, XLOC_ 093758*	*BnaC02g25200D*		
			*XLOC_044363 and XLOC_076449*			
			*XLOC_052298*			
Heat	Wheat	TAM107	*TahlnRNA27, TalnRNA5,*	*–*	Histone acetylation of TalnRNA5	[75]
		Chinease spring	TahlnRNA12, TalnRNA21	*–*		
			*TahlnRNA23 and TahlnRNA29*			
Heat	Chinese cabbage	GHA and XK	*TCONS_00048391*	*NAC*1 (Bra030820)	By targeting bra-miR164a based on	[64]
			*TCONS_00010856*	*Bra021232*	target mimicry mechanism	
			*TCONS_00004594*			
Heat	*Cucumis sativus*	Improved Jinchun 2	*TCONS_00031790, TCONS_00014332,*	–	Interact with miR9748 plant hormone signal	[101]
			*TCONS_00014717,TCONS_00005674*		transduction pathways	
Heat and	*Brassica*		*TCONS_00051908*	–	By acting as targets and eTMs for the miRNAs	[76]
drought	*juncea*		*TCONS_00088973*			
Cold	Cassava	TMS60444	*lincRNA159*	*NAM, ATAF1*/*2,*	Regulate cold tolerance targeting miRNA164	[60]
				*CUC2*	based on target mimicry mechanism	
Cold	*Arabidopsis*	Col-0	*SVALKA*	*CBF1*	SVK represses *CBF1* and	[80]
					increase cold acclimation	
Cold	*Arabidopsis*	Col-0	*COLDWRAP*	*FLC*	*COLDWRAP* reinforc estable	[102]
					repression of *FLC* under cold stress	
Cold	*Arabidopsis*	Col-0	*TAS1a*	–	By alternative spicing of lncRNA	[79]
Cold	*Arabidopsis*	Col-0	*MAS*	*MAF4* gene	Histone modification and role of	[103]
					NAT-lncRNAs regulating gene expression	
Cold	*Brachypodium*	–	*BdCOOLAIR1,BdCOOLAIR2*	*BdODDSOC1,*	BdCOOLAIR transcript represses	[104]
	*distachyon*			*BdODDSOC2*	function of BdODDSOC gene	[104]
Cold	Grapevine	Cabernet Sauvignon	*VIT_203s0017n00360*	Upregulation of the following target	up and down regulation of the target genes	[81]
			*VIT_207s0031n00070*	genes *VIT_216s0100g00380*		
			*VIT_201s0011n00530.*	*VIT_208s0058g00960*		
			*IT_209s0002n00340*	*VIT_215s0046g02110*		
			*VIT_213s0158n00020*	*VIT_202s0025g01280*		
			*VIT_213s0067n00110*	*VIT_200s0246g00150*		
			VIT_200s0225n00020	*VIT_202s0154g00610*		
Cold stress	Chinease cabbage	RJKB-T24	*MSTRG.4795, MSTRG.18513,*	*BrFLC* and *BrMAF* genes	Epigenetic modification at *BrFLC2as* locus,	[82]
			*MSTRG21908,*	related to vernalization	epigenetic modification at *Bra024350* and	
			*MSTRG.259, MSTRG.491*		*Bra031888, Bra024351* and *Bra031884 loci*	
			*MSTRG.17153*			
Cold stress	*Medicago truncatula*	Jemalong A17	*lncRNA MtCIR1*	*MtCBF* genes	Targeting *MtCBF* genes	[84]
Salinity	*Medicago truncatula*	Jemalong A17	lncRNA *TCONS_00097188,*	*Medtr6g006990,*	By regulating various genes	[68]
Salinity			*TCONS_00046739,*	cytochrome P450	related to ROS activity,	
Salinity			*TCONS_00100258*	Transmembrane proteins gene	secondary messenger molecules,	
			and *TCONS_00118328*			
salinity			*TCONS_00047650*	*Medtr3g069280,*	carbonic anhydrase gene etc.	
Salinity			lncRNA *TCONS_00020253*	*Medtr1g081900* and		
Salinity			*TCONS_00116877*	*Medtr7g094600*		
Salinity	*Arabidosis*	–	*DRIR*	ANNAT7,NAC3 and	Affecting fucosyltransferase or	[37]
				*WRKY8*	*NAC3* transcription factor	
Salinity	Cotton	SN91–11	*lnc_388,lnc_883,*	*Gh_A09G1182,*	Targeting *Gh_A09G1182,*	[86]
					*Gh_D03G0339* genes	
			*lnc_973 and lnc_253*	and *Gh_D03G0339* genes	Regulating ghr-miR399 and ghr-156e by eTM	
Salinity	Poplar	*P. euphratica*	*Peu_00167161, Pal_00184400*	*HKT1*	–	
		*P.alba var. pyramidalis*	*Pal_00132209*	fucosyltransferase or *NAC3*		
Salinity	Cotton	SN91–11	lncRNA973	SOD, CAT, POD and P5CS,	lncRNA973 regulate the	[105]
				*RBOHB, RBOHD, NHX7*	ghr-miR399 and its target gene GhPHO2	
				*MYB5, WRKY46, ERF62, NAC29*		
Salinity	*Pistacia vera* L.	*Ghazvini and Sarakhs*	5 NAT-lncRNAs	CERK1, LEA, Laccase genes	NAT-lncRNA regulate ATPase,	[106]
				TF genes, genes related to	cation transporter, kinase	
				hormone signaling pathways	and UDP-glycosyltransferases genes	
Water	Maize	B73	*TCONS_00177501*	*Zm00001d029280*	Assist in water logging tolerance	[92]
logging			*TCONS_00124833*	*Zm00001d012263*		
			*TCONS_00105920*	*Zm00001d015618*		
logging			*TCONS_00092298*	*Zm00001d018819*		
Cadmium	Rice	DX142	*XLOC_086307*	*OS03G0196600*	Genes regulating cysteine and	[88]
				cysteine-rich peptide	methionine metabolism and	
			*XLOC_086119 and XLOC_066284*	metabolism-related gene	carotenoid biosynthesis	
				carotenoid biosynthesis		
Phosphate	*Arabidopsis*		*XLOC_020833, XLOC_001691*	*PHR1*	Regulating phosphate homeostasis	[93]
starvation			*and XLOC_013661*		by targeting miR399	
Phosphate	*Medicago*	Jemalong A17	*PDIL1,PDIL2 and PDIL3*	*MtPHO2*		[61]
starvation	*truncatula*			*Medtr1g074930*		
Nitrogen	Rice	Nipponbare	*cis-NAT*_*AMT1.1*_*,cis-NAT*_*AMT1.2*_	*AMT1.1*and *AMT1.2*	Regulate nitrogen use efficiency	[14]
starvation						
Nitrogen	*Arabidopsis*		*trans-acting siRNA3* (*TAS3*)	*Nitrate transporter 2, NRT2.4*	Maintains cellular N homeostasis by multiple	[99]
starvation				*SEC14p-like protein/Patellin-4*	*tasiRNAs* targeting *ARF2, 3, and 4*	
				*Regulatory component*		
				of ABA receptor 3		
				*PPC3*		
Nitrogen	Barley	Liuzhutouzidamai	*lnc00090* and *lnc000248*	*–*	lnc00090 and lnc000248 target mimics for	[97]
starvation					for hvu-miR399	
Boron	*Poncirus trifoliata*	–	*XLOC_002224*	*Ciclev10008338m*	Targetting *Ciclev10008338m* gene	[98]
deficiecy						

### lncRNAs controlling heat stress tolerance

Heat stress is a major abiotic stress that significantly affects plant growth, physiology, metabolic activity, development, and yield performance [2]. With the current rise in global temperatures, changes in plant phenology and adaptation processes are negatively affecting crop yield, which is challenging global food security [107]. Plants recruit a variety of mechanisms, including adaptive, biochemical, and molecular, to cope with heat stress [2, 108]. Plants produce different phytohormones, heat shock proteins (HSPs)/chaperones, antioxidant enzymes, and metabolites that play a critical role in adjusting to heat stress [108, 109]. At the molecular level, the activation of regulatory pathways plays a role in plant adaptation to heat stress [2]. There is evidence for miRNAs regulating the heat stress response in various plants [110]. The accumulating evidence for lncRNAs acting as an important molecular regulatory layer offers insight into the regulatory mechanism of the heat stress response in crop plants. To explore the role of lncRNAs in conferring a heat stress response, 54 putative heat stress-responsive lncRNAs were identified in wheat using the wheat Affymetrix Gene Chip-based microarray and Solexa sequencing [75]. Among the identified lncRNA transcripts, four and 26 were precursors of miRNAs (viz., miR2004, Ta-miR2010, miR2066) and siRNAs, respectively. Up-regulation of lncRNAs *TahlnRNA27* and *TalnRNA5* and their corresponding miR2010 and miR2004 was confirmed by qRT-PCR analysis, indicating their significant role in the heat stress response in wheat. However, the heat stress response remained tissue-specific/dependent with *TalnRNA5* displaying relatively higher expression in seed tissue than other tissues [75]. Most lncRNAs are weak in sequence conservation; their expression varies from tissue to tissue, developmental stages, and even species to species [65]. Tissue/development-specific expression of lncRNAs has been reported in maize [54, 87] and cassava [53] under drought stress, and species-specific expression was noted for *Populus euphratica* and *Populus alba var. pyramidalis* under salinity stress.

A plethora of differentially expressed lncRNAs and their corresponding protein-coding heat stress-responsive target genes and miRNAs have been identified in various crops [64, 77]. Wang et al. [64] explored the up- and down-regulation of lncRNAs and differentially expressed genes (DEGs) involved in the brassinosteroid, ABA, auxin, jasmonic acid, salicylic acid, and ethylene hormone signaling pathways, and DEGs encoding various heat shock proteins across the whole genome, using strand-specific RNA-seq in *Brassica rapa* under heat stress*.* Among the three identified heat-responsive DElncRNAs, differential expression of lncRNA*TCONS_00004594* downstream at the protein-coding gene *Bra021232* via qRT-PCR suggested its *cis*-regulatory expression [64]. Further, lncRNAs *TCONS_00048391* and *TCONS_00010856* acted as endogenous target mimics for bra-miR164a, which regulates the heat stress response. Consequently, under heat stress, up-regulation of bra-miR164a and down-regulation of lncRNA*TCONS_00048391* and the target *Bra030820* (*NAC1*) gene rendered heat tolerance in ‘XK’ variety ([64], see Fig. 3). Likewise, the binding of *lincRNA159* with conserved miR164 decreased the expression of three miR164-targeted *NAC* genes (*NAM, ATAF1/2, CUC2*) in cassava under cold stress [60]*.* Similarly, drought-responsive lncRNA *MSTRG.42613.1* was identified as the target mimic of conserved miRNA164 regulating drought stress in *C. songorica* [62]. In the future, manipulation of the overexpression or knockout of lncRNAs targeting genes controlling the heat stress response could help us to engineer heat-tolerant crop plants.

### lncRNAs controlling cold stress tolerance

Low-temperature stress is an important abiotic stress that challenges plant growth and yield [2, 111]. Plants orchestrate several complex regulatory gene networks of C-repeat binding factors (*CBFs*) and cold regulated genes (*COR*) [112] and myriad of novel regulatory miRNAs [110] that enable them to acclimate to cold stress. Advances in genetic and genomic approaches have elucidated several QTLs and probable candidate genes contributing to cold tolerance in plants [111]. Likewise, there is emerging evidence of lncRNAs that regulate the cold stress response in plants [65, 80, 103]. The emerging role of lncRNAs regulating cold acclimation is documented in *Arabidopsis* [65, 82], cassava [60], *Brassica rapa* [15, 82], banana, grapevine [81], and Brachypodium [104].

Vernalization is a well-established phenomenon in plant species adapted to cold climates, which prevents flowering during vegetative growth in winter and allows flowering during the reproductive phase under favorable conditions in spring [113]. In *Arabidopsis*, *FLOWERING LOCUS C* (*FLC*) is a well-known regulatory locus that controls flowering time epigenetically [114]. *FLC* also acts as a suppressor of flowering during cold in *Arabidopsis* [115]. In this context, the participatory role of lncRNAs in inhibiting expression of the *FLC* locus by vernalization under cold stress through Polycomb-mediated epigenetic regulation is a well-established mechanism for controlling cold acclimation in *Arabidopsis* [65, 67, 116]. Repression of the *FLC* locus during the early onset of cold stress is controlled by *COLD INDUCED LONG ANTISENSE INTRAGENIC RNAs* (COOLAIR), an alternatively spliced NAT lncRNA transcribed from the antisense orientation of *FLC* gene by chromatin modification (reducing active histone mark H3K36me3 and enhancing repressive histone mark H3K27me3) of the *FLC* locus during vernalization [65–67]. Interestingly, Castaings et al. [117] demonstrated the evolutionarily conserved role of class I antisense COOLAIR that controls *FLC* repression during vernalization in *Arabidopsis thaliana, Arabis lyrata,* and *Arabis alpina* species.

Likewise, *COLD ASSISTED INTRONIC NONCODING RNA* (*COLDAIR*) [116], transcribed from intron1 of the *FLC* gene, recruits the Polycomb Repressive Complex 2 (PRC2) that helps in chromatin modification (increase H3K27me3) of the *FLC* locus and thus represses expression of the *FLC* locus (see Fig. 3). Subsequently, Kim et al. suggested that “Polycomb-binding lncRNA, *COLDWRAP*” could further cooperate in the stable repression of the *FLC* locus during vernalization in *Arabidopsis*.

Recently, Kindergren et al. [80] advanced our understanding of the cold acclimation mechanism in *Arabidopsis* by illustrating the novel role of *SVALKA* and cryptic antisense CBF1 (*asCBF1*) lncRNAs induced by cold stress. These lncRNAs regulate cold acclimation by suppressing transcription of the *CBF1*gene by RNA polymerase II (RNAPII) collision derived from lncRNAs *SVALKA* and *asCBF1*. Likewise, to explore the role of lncRNAs controlling the cold stress response in *Arabidopsis*, strand-specific RNA-sequencing (ssRNA-seq) identified 4050 NAT lncRNAs and 2460 lincRNAs as cold-responsive lncRNAs [103]. Among these, the authors substantiated the novel role of *MAS* (*NAT lncRNA_2962*), a cis-acting NAT lncRNA induced under cold stress, which activated transcription of the corresponding cold-responsive *MADS AFFECTING FLOWERING 4* (*MAF4*)*,* an *FLC* family member, by involving WDR5a complex that deposits H3K4me3 at *MAF4* gene for its activation. Thus, the activated gene eventually suppresses flowering under cold tress. Likewise, in *Brassica rapa,* three *FLC* paralogs that act as a floral repressor during vernalization have been reported [118, 119]. The involvement of NATs at the *FLC2* locus of *Brassica rapa* under cold stress has been reported [120]. RNA-seq driven transcriptome analysis of control and cold-treated leaves of *Brassica rapa* identified 2088 lncRNAs [82], of which three *BrFLC* loci contributed to cold stress regulation—only *BrFLC2,* harboring NAT *BrFLC2as* (*MSTRG.2765*), had homology to the COOLAIR transcript of *Arabidopsis thaliana* and displayed up-regulation under cold stress [82]. Functionally, COOLAIR acts as “*cis*-NAT with respect to the *AtFLC* locus” [67]; however, the action of *BrFLC2as* as *cis-* or *trans-*acting mode needs further investigation. Likewise, considering the role of the *MAF* gene, the *Bra024350* locus (homologous to *AtMAF1*)—with a NAT known as *MSTRG.14523*—was down-regulated under cold stress. However, the *Bra024351* locus (homologous to *AtMAF4*)—with a NAT known as *MSTRG.14524*—was not down-regulated under cold stress in *Brassica rapa*, suggesting that the working mechanism of the lncRNAs mentioned above differed from the lncRNAs involved in vernalization in *Arabidopsis thaliana* [82]. Furthermore, among the plethora of differentially expressed lincRNAs, NAT lncRNAs identified lncRNAs *MSTRG.4795, MSTRG.18513,* and *MSTRG21908* as up-regulated and *MSTRG.259, MSTRG.491,* and *MSTRG.17153* as down-regulated under cold stress imposed at various stages in *Brassica rapa* [82].

A genome-wide survey for cold-responsive lncRNAs in grapevine using RNA-seq analysis recovered 284 novel up-regulated lncRNAs, 182 novel down-regulated lncRNAs, 242 DElncRNAs targeting 326 protein-coding genes, and various stress-responsive genes including *CBF4* transcription factor genes, late embryogenesis abundant protein genes, and *WRKY* transcription factor genes [81]. Functional validation of selected lncRNAs through qRT-PCR confirmed up-regulation of lncRNAs *VIT_200s0179n00030, VIT_207s0141n00070*, and *VIT_207s0005n0048* and down-regulation of *VIT_201s0010n00070*, *VIT_208s0007n00270,* and *VIT_209s0002n00020,* suggesting their important role in regulating cold stress tolerance in grapevine [81]. In cassava, to unveil cold and drought-responsive lncRNAs genome-wide, 318 lncRNAs were captured [60]. Considering their contributory role in cold stress tolerance, functional validation of *lincRNA419, 207,* and *234* revealed their up-regulated activity under cold stress. To decipher the regulatory network of miRNAs, lncRNAs, and the stress-responsive gene controlling cold tolerance, *lincRNA159* acting as target mimic for miR164 decreased the expression of *NAC* genes under cold stress [60]. Apart from these mechanisms, alternative splicing (AS) of lncRNAs and pri-miRNAs could participate in controlling the cold stress response in *Arabidopsis* [79]. Of the 135 lncRNAs identified with cold-dependent differential expression and differential alternative splicing, induction of *TAS1a* lncRNA regulated by AS under cold stress was uncovered in *Arabidopsis*. The unspliced intron-containing transcript AT2G27400.1 produced from *TAS1a* contained “miR173binding site and *tasiRNAs* generation site” while the spliced transcript AT2G27400_ID1 remained intronless. Given the decrease in temperature, the AT2G27400_ID1 transcript decreased rapidly in the first 6 h after cold treatment, whereas unspliced AT2G27400.1 increased in the first 3 h. Subsequently, it declined over the next 12 h [79]. Thus, AS of lncRNAs plays an important role in regulating cold stress tolerance. LncRNAs could regulate cold tolerance through chromatin modulation/remodeling, AS mechanisms, and transcriptional regulation of genes contributing to cold tolerance. Further understanding of the working mechanism of lncRNAs controlling cold stress may provide opportunities for engineering cold-tolerant crops.

### lncRNAs as new players in plant acclimation under salinity stress

The indiscriminate practice of excessively irrigating farmland and the rapid depletion of groundwater are major factors associated with the increase in salinity-related problems worldwide [121]. Globally, 45 Mha of irrigated land and 32 Mha of hardy land are challenged by salinity stress [122, 123]. Thus, soil salinization remains an increasing constraint to global food production. Under salinity stress, plants suffer from an excessive load of toxic ions, which reduces plant growth and development and grain yield [124].

Plants have evolved several cellular and physiological mechanisms to adapt to salinity stress (see [124]). At the molecular level, a plethora of ion transporter proteins encoded by gene(s)/QTLs and other regulatory genes play a crucial role in controlling salinity stress in various plants (see [121, 124]). Likewise, evidence of regulatory roles of lncRNAs enabling plants to tolerate salinity stress has advanced our understanding of the molecular mechanisms controlling the salinity stress response in plants [37, 55].

To elucidate the functional role of lncRNAs in *Medicago truncatula* under salinity stress and osmotic stress, several lncRNAs have been identified, including *TCONS_00046739* (regulating cytochrome P450 in roots), *TCONS_00097188* (regulating photosynthesis by up-regulating *Medtr6g006990* gene), *TCONS_00047650* (up-regulating expression of the *Medtr3g069280* gene encoding phosphatidylinositol-specific phospholipase C), *TCONS_00116877* (up-regulating the *Medtr7g094600* gene encoding glutathione peroxidase in roots), and *TCONS_00020253* (up-regulating expression of Na^+^/H^+^ exchanger gene *Medtr1g081900* in roots) ([68], see Fig. 3). Likewise, the presence of lncRNA *DRIR* regulates higher expression of *P5CS1*, *RD29A*, *RD29B*, *AtrbohB*, *FUT4*, *ANNAT7*, and *NAC3* genes that confer salinity stress tolerance in the *drir*^*D*^ mutant and *DRIR*-overexpressing lines in *Arabidopsis* [37]. In cotton, deep transcriptome sequencing of salt-treated leaf tissue facilitated the identification of 44 differentially expressed lincRNAs from 1117 unique lncRNAs [86]. Functional validation of selected lincRNAs via RT-qPCR revealed the up-regulatory role of *lnc_388* on *cis-acting* target leucine-rich repeat 8 (*Gh_A09G1182*) gene and *lnc_883*lncRNA targeting on Gh_D03G0339MS_channel protein-coding gene under salinity stress (Table 2). The authors also confirmed the role of lncRNAs *lnc_973* and *lnc_253* acting as target mimics for ghr-miR399 and ghr-156e under salinity stress [86]. Likewise, 1710 lncRNAs that were responsive to combined salinity and boron stress were explored in the Lluteño landrace of maize using deep transcriptome analysis of leaf and root tissue-derived RNA libraries [87]. Interestingly, a comparison of the genome sequences of three popular maize cultivars (B73, Mo17, and Palomero) and the Lluteño landrace identified the presence of 955 conserved lncRNA transcripts; however, 755 were exclusive to the Lluteño landrace, which may explain its salinity and boron stress tolerance [87]. To gain insight into the salinity and boron response of lncRNAs, functional validation of 12 trans-NAT lncRNAs from 848 differentially expressed trans-NAT lncRNAs suggested their significant role in controlling various stress regulatory gene expression, including combined salinity and boron stress and the nicotinamide metabolic process [87]. Thus, the identified lncRNAs conferred salinity stress tolerance by controlling oxidative stress through modulating genes encoding for antioxidant enzymes and regulating various Na^+^/H^+^ exchanger genes and other regulatory genes related to salinity stress.

### lncRNAs regulating nutrient deficiency in plants

Nutrient acquisition from soil remains one of the essential physiological processes for regulating plant growth and development [125]. Several molecular mechanisms, including many nutrient transporters, are actively involved in plant nutrient homeostasis [126, 127]. Among the various non-coding regulatory RNAs, evidence of miRNAs and lncRNAs regulating nutrient acquisition has been found in various plants [11, 14, 61].

Among the major nutrients, phosphorus (P) serves as a fundamentally important element contributing to plant growth and development; it also acts as a P source for ATP production [128]. The availability of soil inorganic phosphate (Pi) to plants is constrained by several factors that limit overall plant growth and development [129]. Little information is available on the complex regulatory network of P homeostasis in plants [127, 130]. Several molecular and biochemical mechanisms are activated by plants to improve soil inorganic phosphate availability and increase phosphorus use efficiency (PUE) [127, 129, 130]. In this context, the role of miRNAs controlling phosphate availability has been reported in various plants [110]. Likewise, the emerging role of lncRNAs regulating phosphate content in plants is well-established in *Arabidopsis* [11, 93], rice [14, 131], and the model legume *Medicago truncatula* [61]. The working mechanism of miR399 and its target gene *PHOSPHATE2* (*PHO2*) is well-recognized for regulating phosphate content in *Arabidopsis* [132, 133]. Subsequently, Franco-Zorrilla et al. [11] revealed the inductive mechanism of lncRNA *INDUCED BY PHOSPHATE STARVATION 1*(*IPS1*) that works as an eTM or decoy for miR399 and regulates the target *PHO2* gene expression and phosphate homeostasis in *Arabidopsis*. Thus, given the abundance of phosphate, lncRNA *IPS1* binds to miR399 and prevents it from acting on target gene *PHO2*, which presumably abolishes the functional role of phosphate transporters by the ubiquitination pathway, thereby restricting root uptake of excessive phosphate ([69], see Fig. 3). Under phosphate-deficient conditions, the *PHO2* gene is suppressed as miR399 degrades the transcript of *PHO2* and eventually allows phosphate transporters to accumulate phosphate [132].

Furthermore, the phosphate regulation mechanism—based on the “*PHR1*–miR399–*PHO2”* pathway in association with phosphate deficiency-responsive lncRNA*PDIL1,* a paralog of *Mt4—*has been demonstrated in *Medicago truncatula* [61]. The authors established a negative regulatory role of the lncRNAs*PDIL2* and *PDIL3* controlling the expression of the phosphate transporter gene *Medtr1g074930.* Likewise, the working mechanism of *cis-NATPHO1;2* lncRNA functioning as a translational enhancer of the *PHO1;2* gene for phosphate homeostasis has been reported in rice [131].

Like P, nitrogen (N) is an essential nutrient for plant growth and development, and also serves as an N source for amino acids, ATP, and N metabolism in plants [134]. Several QTLs in various crops of agricultural importance reportedly improve nitrogen use efficiency (NUE) [134]. Advances in functional genomics approaches have identified several regulatory gene(s) and transporter genes controlling NUE in crop plants [135]. However, the entire molecular mechanism of N assimilation is not understood in plants [136]. State-of-the-art deep transcriptome sequencing via RNA-seq has further advanced our understanding of N-responsive lncRNAs contributions to N homeostasis in plants. Numerous N-responsive lncRNAs have been uncovered in various plant species viz., rice, maize, poplar [14, 95, 96]. The operating mechanism of lncRNAs *cis-NAT*_*AMT1.1*_ and *cis-NAT*_*AMT1.2*_, targeting the *AMT1* gene for N homeostasis, is well-recognized in rice [14]. A study on lncRNAs in the *Arabidopsis* genome under various nutrient-deficient conditions uncovered the role of *trans-acting siRNA3* (*TAS3*) as an important lincRNA targeting the *nitrate transporter 2* gene, thereby regulating N transport in N-starved environments [99].

Among the various micronutrients, boron (B) is an essential micronutrient for plant growth and development, membrane integrity, and cell wall synthesis [137–139]. Genome-wide exploration of lncRNA regulating B deficiency response in *Poncirus trifoliata* through strand-specific deep transcriptome analysis detected 2101 unique lncRNAs [98]. Further, expression profiling analysis identified 729 up-regulated and 721 down-regulated lncRNAs under B deficiency stress. Functional validation of selected lncRNAs shed light on the target genes involved in the calcium signaling and plant hormone signal transduction pathways under B deficiency stress in *Poncirus trifoliata* [98].

The above findings have laid the foundation for future in-depth research on the regulatory role of various lncRNAs controlling nutrient deficiency in plants.

### Role of lncRNAs under heavy metal toxicity

The outcome of rapid industrialization, application of heavy doses of chemical fertilizers, and indiscriminate contamination of heavy metals in irrigation water and arable land have posed a serious challenge for crop yields and human health [140], particularly cadmium. To minimize heavy metals moving from the soil into plants, plants use several regulatory molecular mechanisms [140]—lncRNAs may play a crucial role in controlling the uptake of heavy metals into the plant system.

RNA-seq profiling identified 301 cadmium-responsive lncRNAs in *Brassica napus*, of which 67 were eTMs for 36 Cd-responsive miRNAs [141]. Functional validation of *TCONS_00091906, TCONS_00033487*, and *TCONS_00097191* lncRNA under Cd stress using qRT-PCR analysis indicated their significant role as target mimicry for EL628609, TC182597, and TC203372 mRNAs involved in Cd uptake and detoxification [141]. Likewise, Chen et al. [88] undertook a genome-wide survey of lncRNAs using RNA deep transcriptome sequencing that provided evidence of both up- and down-regulation of lncRNAs involved in the Cd response. Furthermore, functional analysis of DElncRNA provided insight into the role of lncRNAs regulating target genes associated with cysteine and methionine metabolism under Cd stress (see Fig. 3). Considering the mounting evidence of arsenic (As) toxicity in rice, Tang et al. [142] provided novel insights into As-responsive lncRNAs along with other non-coding RNAs regulating the As toxicity response in rice. However, the mechanisms involved in the regulatory role of lncRNAs controlling heavy metals is unknown and needs further research.

### Database and web-based resources of lncRNAs

Advances in functional genomics, especially RNA-seq analysis, have enabled the discovery of novel lncRNAs that regulate various biological processes, including stress responses. However, the accurate prediction of lncRNAs, their structure, genomic content, conservation, and functional annotation remains a challenge (see [8]). To address these shortcomings, several web-based resources and databases have been developed, viz., NONCODE provides the comprehensive biological functions of lncRNAs [143–145], PLNlncRbase contains information on 1187 plant lncRNAs from more than 40 species [146], and Plant Long non-coding RNA Database (PLncDB) offers information on 6480 lncRNAs in *Arabidopsis* [147]. Likewise, the Plant Natural Antisense Transcripts Database (PlantNATsDB) provides information on plant NATs controlling various physiological and development processes [148], Plant ncRNA Database (PNRD) maintains records of 25,739 non-coding RNAs including lncRNAs [149], CANTATAdb maintains 45,117 lncRNAs from 10 plant species [16], CANTATAdb 2.0. annotates plant lncRNAs [155] and PLncPRO provides information on abiotic stress-responsive lncRNAs in rice and chickpea [72]. A detailed list of plant lncRNA databases is in Table 3. Several important tools, such as CPPred [158], REPTree [159], Pfamscan [160], COME [161], PLIT [156], and CPC2 [162], are available to distinguish lncRNAs from mRNAs. Advances in bioinformatics tools and new algorithms could further boost our efforts in discovering novel lncRNAs and their accurate functional annotations.

**Table 3:** Databases of various lncRNAs obtained in plant species

Name	Characteristics	lncRNA and details	References	Link
PLncDB	It provides comprehensive data on Arabidopsis lncRNAs	Arabidopsis lncRNAs	[147]	http://chualab.rockefeller.edu/gbrowse2/homepage.html
PLNlncRbase	Detailed information on experimentally identified plant lncRNAs	Supply information on 1187 plant lncRNAs in	[146]	http://bioinformatics.ahau.edu.cn/PLNlncRbase/
		43 plant species		
PNRD	It provides information on different types of ncRNAs	150 plant species	[149]	http://structuralbiology.cau.edu.cn/PNRD
CANTATAdb	Used for annotation of identified lncRNAs	Covers information on lnc RNA on 10 plant species	[16]	http://cantata.amu.edu.pl, http://yeti.amu.edu.pl/CANTATA/
GREENC	Used for annotate lncRNAs	Annotation of more than 120,000 lncRNAs associated to 37 plant species could be done	[150]	http://greenc.sciencedesigners.com/
PLncPRO	Used for prediction of lncRNAs in plants and used fornvestigating abiotic stress responsive lncRNAs in rice and chickpea	3714 and 3457 lncRNAs in rice and chickpea for drought and salinity	[72]	http://ccbb.jnu.ac.in/plncpro.
PlaNC-TE	Provide insights about the relationship between ncRNA and TEs in plants	Information on overlapping of ncRNA and transposon elements from 40 plant genomes	[151]	http://planc-te.cp.utfpr.edu.br
EVLncRNAs	It contains lncRNA information on various species including plant	1543 lncRNAs from 77 species and also 428 plant lncRNAs from 44 plant species	[152, 153]	http://biophy.dzu.edu.cn/EVLncRNAs.
CRISPRlnc	Database for validated CRISPR/Cas9 sgRNAs for lncRNAs from variousspecies including plants	305 lncRNAs and 2102 validated sgRNAs on eight species including plant	[154]	http://www.crisprlnc.org or http://crisprlnc.xtbg.ac.cn
CANTATAdb 2.0	It provides information on annotation of plant lncRNAs	Covers information on lnc RNA on 39 plant species	[155]	http://cantata.amu.edu.pl, http://yeti.amu.edu.pl/CANTATA/
PLIT	Used for investigating of plant lncRNAs from RNA seq data.	Provides information on lncRNA from 8 plant species	[156]	
PLncDB	Detail information on plant lncRNAs	Provides plant lincRNAs and lncNATs information	[157]	–

The table is updated version of [17, 61, 143]

## Conclusion

The rapidly increasing number of plant lncRNAs and their multifaceted regulatory roles in governing various biological processes is becoming a hotspot in biological research [8, 12]. However, genome-wide discovery, characterization, and functional annotation of lncRNAs remain limited in plant species. The increasing availability of reference genome sequences of crop plants could offer opportunities to explore various lncRNAs and their sequence similarity and ‘functional conservation’ using comparative genome analysis [38]. Further, in-depth transcriptome sequencing, rapid advances in computational biology, and increasing databases for lncRNAs and efficient methods/tools could assist in the prediction of accurate lncRNAs and functional annotation of novel lncRNAs. The paucity of mutants corresponding to lncRNAs is another challenge for functional analysis of novel lncRNAs [17]. In this context, CRISPR/Cas9 engineered mutation in novel abiotic stress-responsive lncRNAs could shed light on the function of lncRNAs, and thus help in the design of abiotic stress-tolerant crop plants [163]. The availability of a comprehensive atlas of lncRNAs across whole genomes in crop plants, coupled with a comprehensive understanding of the complex molecular mechanisms that regulate various abiotic stress responses, will enable us to use lncRNAs as potential biomarkers for tailoring abiotic stress-tolerant plants in the future.

## Data Availability

Not applicable.

## Abbreviations

lncRNA: Long non-coding RNA
miRNA: MicroRNA
ncRNAs: Non-coding RNAs
RNA-seq: RNA-sequencing
siRNAs: Small interfering RNAs
eTM: Endogenous target mimics
lincRNAs: Long intergenic lncRNAs
NAT: Natural anti-transcript
DElncRNAs: Differentially expressed lncRNAs
v*pp4*: *V-ATPase encoding gene*
*DRIR*: *DROUGHT INDUCED lncRNA*
DEGs: Differentially expressed genes
*CBFs*: *C-repeat binding factors*
*COR*: *Cold regulated genes*
*FLC*: *FLOWERING LOCUS C*
*COOLAIR*: *COLD INDUCED LONG ANTISENSE INTRAGENIC RNAs*
*COLDAIR*: *COLD ASSISTED INTRONIC NONCODING RNA*
RNAPII: RNA polymerase II
P: Phosphorus
N: Nitrogen
B: Boron
*IPS1*: *INDUCED BY PHOSPHATE STARVATION 1*
NUE: Nitrogen use efficiency
